# Exploring the physiological correlates of chronic mild traumatic brain injury symptoms

**DOI:** 10.1016/j.nicl.2016.01.004

**Published:** 2016-01-06

**Authors:** Serguei V. Astafiev, Kristina L. Zinn, Gordon L. Shulman, Maurizio Corbetta

**Affiliations:** Department of Neurology, Washington University in St. Louis, 660 S. Euclid Ave, Campus Box 8225, St. Louis, MO 63110, USA

**Keywords:** Traumatic brain injury, MRI, Diffusion tensor imaging, Behavioral assessments

## Abstract

We report on the results of a multimodal imaging study involving behavioral assessments, evoked and resting-state BOLD fMRI, and DTI in chronic mTBI subjects. We found that larger task-evoked BOLD activity in the MT+/LO region in extra-striate visual cortex correlated with mTBI and PTSD symptoms, especially light sensitivity. Moreover, higher FA values near the left optic radiation (OR) were associated with both light sensitivity and higher BOLD activity in the MT+/LO region. The MT+/LO region was localized as a region of abnormal functional connectivity with central white matter regions previously found to have abnormal physiological signals during visual eye movement tracking (Astafiev et al., 2015). We conclude that mTBI symptoms and light sensitivity may be related to excessive responsiveness of visual cortex to sensory stimuli. This abnormal sensitivity may be related to chronic remodeling of white matter visual pathways acutely injured.

## Highlights

• We report a multimodal imaging study of symptoms in chronic mTBI.
• Higher BOLD activity in the MT +/LO region correlated with mTBI symptoms.
• Higher FA near the left optic radiation was associated with light sensitivity.

## 1 Introduction

The Centers for Disease Control and Prevention (CDC) estimate that each year approximately 2.5 million Americans sustain a traumatic brain injury (Faul et al., 2010). Traumatic brain injuries can be classified into mild, moderate, and severe categories, and about 90% of all TBI cases in USA are classified as mild TBI (mTBI) (Narayan et al., 2002).

Mild TBI is associated with a host of symptoms and signs: headache, confusion, lightheadedness, dizziness, blurred vision or tired eyes, ringing in the ears, fatigue or lethargy, a change in sleep patterns, behavioral or mood changes (including posttraumatic stress disorder (PTSD) and depression), and problems with memory, concentration, and attention (Chen, A.J. and D'Esposito, M., 2010, Gerber, D.J. and Schraa, J.C., 1995, Ghajar, J., 2000, Kushner, D., 1998, McAllister, T.W., 2011, McDowell, S., et al., 1997). It is unknown why a percentage of mTBI individuals (**~** 10–15% of adults, and up to 40% of children) (Crooks, C.Y., et al., 2007, Dikmen, S., et al., 1986, McCrea, M., et al., 2009, Thornhill, S., et al., 2000) continue to manifest symptoms at the chronic stage. Conventional structural imaging scans are typically normal.

Many studies suggest that mTBI symptoms largely overlap with symptoms of PTSD and depression (Barnes, S.M., et al., 2012, Bryant, R., 2011, Hoge, C.W., et al., 2008, Mac Donald, C.L., et al., 2014, McKee, A.C., et al., 2013, Schneiderman, A.I., et al., 2008). A recent study (Hoge et al., 2008) found that adjusting for PTSD and depression symptoms eliminated the association between mTBI and most physical symptoms (except for headache) in U.S. soldiers, indicating the importance of determining the overlap between depression, PTSD and mTBI symptoms. Another recent study (Mac Donald et al., 2015) demonstrated that a diagnosis of traumatic brain injury, older age, and more severe post-traumatic stress symptoms predicted poor outcome after military mTBI. Similarly, another study from the same group (MacDonald et al., 2014) demonstrated high rates of PTSD and depression 6–12 months after concussive blast-plus-impact complex TBI One hypothesis is that chronic mTBI symptoms are in large part psychosomatic, representing a learned pattern of behavior that partly relates to depression and PTSD.

In a previous paper (Astafiev et al., 2015) we demonstrated that symptomatic chronic mTBI subjects show abnormal brain activation during visual tracking tasks. Abnormal activity measured with blood oxygenation level dependent (BOLD) functional magnetic resonance imaging (fMRI) occurred in a common set of subcortical and white matter regions including the anterior internal capsule (IC) and superior longitudinal fasciculus (SLF) white matter pathways that have been previously identified as particularly susceptible to trauma (Bendlin, B.B., et al., 2008, Huisman, T.A., et al., 2004, Laitinen, T.P., et al., 2009, Lipton, M.L., et al., 2008, Niogi, S.N., et al., 2008). In contrast, normal responses were observed in frontal eye field and intraparietal sulcus, cortical regions that are commonly recruited during eye movements and visual attention. The abnormal BOLD signals in the subcortical regions and white matter accurately differentiated chronic mTBI patients from healthy controls at the level of single subjects (linear discriminant analysis accuracy using ‘leave-one-out’ cross-validation was 78.4%), but did not correlate with symptoms or neuropsychological performance. We proposed that the abnormal BOLD signals might reflect structural remodeling secondary to acute injury.

In this paper, we test the hypothesis that remodeled regions in the white matter or subcortical nuclei are abnormally connected with other cortical regions, and that activity in abnormally connected cortical regions will relate to mTBI symptoms. Currently, no consistent brain mechanism or localization has been found that explains mTBI symptoms (Barnes, S.M., et al., 2012, Hoge, C.W., et al., 2008, Mac Donald, C.L., et al., 2014, Mayer, A.R., et al., 2015, McKee, A.C., et al., 2013, Schneiderman, A.I., et al., 2008). Our hypothesis is quite novel as it starts from a physiological hypothesis about the origin of mTBI symptoms, which is consistent with findings of abnormal cortical dynamics in severe TBI (Palacios, E.M., et al., 2013, Rigon, A., et al., 2015, Venkatesan, U.M., et al., 2015), as well as in other diseases of the white matter, e.g. multiple sclerosis (Hawellek, D.J., et al., 2011, Rocca, M.A., et al., 2015).

To localize cortical regions with abnormal connectivity we employed resting state functional connectivity MRI (rs-fcMRI or FC), a method sensitive to the statistical co-variation of activity between brain regions (Biswal et al., 1995). Rs-fcMRI has been shown recently to detect directional gradients in the white matter approximating those measured with DTI (Ding et al., 2013). By seeding the subcortical/white matter regions with abnormal BOLD responses during eye tracking in (Astafiev et al., 2015), we identified one region in extra-striate visual cortex (Middle Temporal/Lateral Occipital: MT+/LO) that was abnormally connected in mTBI. Activity in this region during eye tracking was abnormally high in mTBI and related to symptoms, especially headaches and light sensitivity. Moreover, we found that DTI in the optic radiation was abnormal, and predicted both BOLD signals in MT+/LO mTBI symptoms. Our findings therefore support the hypothesis that symptoms in mTBI are due to abnormal activity in abnormally connected cortical regions.

## 2 Materials and methods

### 2.1 Subjects

Twenty chronic mTBI patients (9 males) and twenty-two age and education-matched normal control subjects (10 males) participated. All mTBI patients were diagnosed with mTBI at the Washington University School of Medicine Concussion clinic. All patients reported post-traumatic amnesia (PTA), and 94% of subjects reported loss of consciousness (LOC) (see Supplemental Table S1 in (Astafiev et al., 2015) for detailed description of the subjects). Informed consent was obtained in accordance with procedures approved by the local human studies committee.

Based on the literature (Boake, C., et al., 2005, Kovesdi, E., et al., 2010, Leddy, J.J., et al., 2010, McCrea, M., et al., 2009, Yuh, E.L., et al., 2013), we defined mTBI as chronic after 3 months post injury. The inclusion criteria were as follows: isolated traumatic brain injury with or without loss of consciousness (LOC) between 3 months to 5.5 years prior to testing, any persistent post-concussive symptoms, any length of post-traumatic amnesia (PTA), Glasgow Coma Scale (GCS) of 13–15 at time of injury, age 18–60. Exclusion criteria for both controls and mTBI patients were as follows: neurological or pre-morbid psychiatric disorders (including ADHD and seizure disorder), alcohol (ETOH)/substance abuse, gross visual (worse than 20/30 corrected) or hearing problems, metal objects in body (except objects that are proven to be safe for 3T MRI), pregnancy and severe claustrophobia.

Informed consent was obtained in accordance with procedures approved by the local human studies committees. Normal control subjects were recruited from the university's database of healthy research volunteers. They were not related to the mTBI subjects and were matched for age and education. Control subjects were required to have no history of TBI, closed head injury, or concussion as confirmed by BISQ (Brain Injury Screening Questionnaire) as well as depression and PTSD (Post-Traumatic Stress Disorder).

Behavioral data from one chronic mTBI patient were incomplete. One control subject was not able to participate in the imaging sessions due to claustrophobia. Two mTBI subjects withdrew from the imaging study, and one was removed due to excessive movement. One healthy subject did not participate in the imaging session due to claustrophobia and one was removed because of excessive movement. Therefore, 17 chronic mTBI patients and 20 control subjects were included in the final analysis of the fMRI scans of the visual tracking tasks.

### 2.2 Neuropsychological testing

The following neuropsychological tests were administered: Head Injury Symptom Checklist (HISQ), Brain Injury Screening Questionnaire (BISQ), Center for Epidemiologic Studies Depression Scale (CES-D), PTSD CheckList — Civilian Version (PCL_C) among others (see Astafiev et al. (2015) for the detailed description). If normative data were available, raw neuropsychological scores were transformed to standardized scores; otherwise, we transformed the raw scores into z-scores based on the healthy controls and mTBI patient samples. Post-concussive symptoms were measured using the HISQ 1-20. The results of several tests: Head Injury Symptom Checklist (HISQ), Conners Center for Epidemiologic Studies Depression Scale (CES-D), PTSD Checklist (PCL_C) were reported as raw scores for the lack of normative scores.

### 2.3 Visual tracking tasks

The detailed description and analysis of the visual tracking tasks was presented in a previous report (Astafiev et al., 2015), so they are only briefly described in this paper. The pursuit target was a red disk moving clockwise in a circular trajectory with a radius of 6° at 0.4 Hz. Three different smooth-pursuit tracking tasks were used:

During a ‘Tracking Alone’ (TA) task, only the target was presented on screen, as in Maruta et al. (2010). During a ‘Tracking with Distracters’ (TD) task, a distracter disk (a red disk identical to the target, but moving with a slightly different circular trajectory) was occasionally presented for 800–1200 ms with a random ISI of 800–1500 ms. The distracter phase angle always crossed the target phase angle (i.e. the path of the distracter either fell behind or moved ahead of the target), although the target and distracter stimuli always remained distinct throughout the trajectory because of their different radial distances or eccentricities. This task was designed to measure a patient's ability to suppress distracting information, a frequent complaint of mTBI patients.

During a Gap (GAP) task the target sometimes disappeared. The subject was instructed to follow the target's movement as closely as possible and anticipate the target's movement if it was not visible. Three gap durations were randomly presented: short: 30° (208 ms); medium: 45° (312 ms); long: 60° (416 ms) with random ISI of 1250–3250 ms. This task was designed to measure a patient's ability to maintain predictive signals, which are necessary for accurate tracking, in the absence of sensory input (Diwakar et al., 2015).

During all 3 tasks, a central red dot was presented when the target was not moving (fixation-only periods). Each eye movement task was performed in three separate scans, where each scan lasted for 2.8 min. The order of scans was determined using a Latin square. Each trial of tracking lasted 15 s, consisting of six 2.5 s cycles, and was followed by a fixation period (only central red fixation dot presented) of 9 s, 11 s or 13 s, randomly determined. A random fixation interval allowed us to estimate the BOLD signal during the visual tracking task without assuming a hemodynamic response function (Boynton, G.M., et al., 1996, Ollinger, J.M., et al., 2001). Six trials were presented within each scan. Before each MRI scan started, the name of the task was visually presented on screen for several seconds, disappearing with the start of first MRI frame and replaced by the central red fixation dot, which was presented for 8 s. Eye movements were recorded in all subjects. A nine-point eye position calibration was performed before each block in all sessions.

### 2.4 Apparatus

An infrared eye-tracker (EyeLink 1000, SR Research Ltd., Ontario, Canada) was used to record eye movements binocularly in the behavioral session (sampling at 500 Hz) and monocularly during the MRI sessions (sampling at 500 Hz). A desktop mount with chin rest was used in the behavioral session, and a long-range mount with head stabilization was used in the MRI session.

Stimuli were generated on a PC running Windows XP (Microsoft, WA, USA) and using Experiment Builder (SR Research Ltd., Ontario, Canada), which allowed online integration with the EyeLink 1000 (SR Research Ltd., Ontario, Canada) eye tracker. Visual stimuli were presented during the behavioral session on a Samsung SyncMaster 2233RZ (Samsung, NJ, USA) LCD monitor (1680 × 1050 @ 120 Hz; see (Wang and Nikolic, 2011)), during the imaging session on a Boxlight CD715X (Boxlight Corporation, WA, USA) DLP projector (1024 × 768 @ 75 Hz) and rear projection screen.

### 2.5 MRI imaging sessions

All scans were collected on a Siemens 3T Tim-Trio scanner. Structural scans included a sagittal MPRAGE (magnetization-prepared rapid acquisition gradient echo) T1-weighted image; TR = 1950 ms, TE = 2.26 ms, flip angle = 9°, voxel size = 1.0 × 1.0 × 1.0 mm) and a transverse turbo spin-echo T2-weighted image (TR = 2500 ms, TE = 435 ms, voxel-size = 1.0 × 1.0 × 1.0 mm).

BOLD contrast (for both task-evoked data and rs-fcfMRI) was measured with a gradient echo EPI sequence (TR = 2000 msec, TE = 27 ms, 32 contiguous 4 mm slices, 4 × 4 mm in-plane resolution). Preprocessing consisted of the following steps: 1) asynchronous slice acquisition was compensated by sinc interpolation to align all slices; 2) elimination of odd/even slice intensity differences resulting from interleaved acquisition; 3) a whole brain normalization corrected for changes in signal intensity across scans; 4) data was realigned within and across scans to correct for head movement; 5) EPI data was co-registered to the subject's T2-weighted anatomical image, which in turn was co-registered with the T1-weighted MP-RAGE, in both cases using a cross-modal procedure based on alignment of image gradients (Rowland et al., 2005). The MP-RAGE was then transformed to an atlas-space (Talairach and Tournoux, 1988) representative target using a 12-parameter affine transformation. Movement correction and atlas transformation was accomplished in one resampling step (resulting in an isotropic 3 mm voxel size) to minimize blur and noise. The first four frames of each scan were eliminated to allow steady-state magnetization, and the remaining frames were concatenated. In preparation for the rs-fcfMRI analysis, data were passed through several additional preprocessing steps previously described (Baldassarre, A., et al., 2014, Fox, M.D., et al., 2005): (1) spatial smoothing (6 mm full width at half maximum Gaussian blur in each direction); (2) temporal filtering retaining frequencies in the 0.009–0.08 Hz band; (3) removal of the following sources of spurious variance unlikely to reflect spatially specific functional correlations through linear regression: (i) six parameters obtained by rigid body correction of head motion, (ii) the whole-brain signal averaged over a fixed region in atlas space, (iii) signal from a ventricle, and (iv) signal from a white matter region.

DTI scans consisted of two averages of a 64-direction diffusion tensor imaging sequence (voxel size = 2 × 2 × 2 mm; TR = 9200 ms; TE = 92 ms; 9 × b-value = 0 s/mm^2^; the rest b-value = 1000 s/mm^2^. Similar to the BOLD data, DTI data were preprocessed and transformed into standardized Talairach atlas space. Co-registration of each DTI image set was performed using vector gradient measure (VGM) maximization (Rowland et al., 2005). The first acquired, unsensitized (b = ~ 0 s/mm^2^) DTI volume was registered to the T2 image; stretch and shear was enabled (9-parameter affine transform) to partially compensate for subject motion and eddy current distortion. T2 then was co-registered similarly to T1, which was registered into standardized Talairach atlas space (Talairach and Tournoux, 1988). We conducted voxelwise analyses in which the BOLD magnitudes in a seed ROI were correlated with FA (fractional anisotropy) values in white and gray matter. Results of voxel-wise statistical tests were corrected for multiple comparisons (Monte Carlo correction; cluster size of 53 voxels with z ≥ 2.25). In order to analyze only the white matter voxels inside an ROI, we selected voxels that had an FA value of 0.2 or higher.

We collected structural scans, DTI scans plus 7 rs-fcfMRI scans (128 frames per scan; 4.3 min duration) during the first imaging session and 9 task scans (83 frames per scan; 2.8 min duration), in which subjects performed the ocular pursuit tasks inside the MRI scanner, during the second imaging session. The mean time interval between the two sessions was 21.4 (SD = 28.5) days for mTBI patients and 9.9 (SD = 9.03) days for control subjects. There were no significant differences in time interval between the two sessions for TBI patients and controls. All frames in rs-fcfMRI scans (imaging session 1) with DVARS (temporal derivative of timecourses of RMS variance over voxels) value of 3.7 or higher were removed from the analysis. DVARS indexes the rate of change of the BOLD signal across the entire brain at each frame of data; DVARS was calculated as described in Power et al. (2012). To define the DVAR threshold for our group of subjects, we computed mean plus 2SD of DVARS values for all frames, excluding first 4 frames in the group of matched control subjects. In addition, all functional MR frames in the imaging session involving ocular pursuit tasks with a total head movement score of 0.9 mm or higher, including the frame immediately after the frame that exceeded the movement threshold, were removed from the analysis. Head movement values were calculated by differentiating head realignment parameters across frames (which yielded a six dimensional timeseries that represents instantaneous head motion) and converting them to a single number using a previously-published method (Power et al., 2012).

The blocked-design task scans were analyzed using an assumed hemodynamic response function (HRF) (Boynton et al., 1996) within the general linear model (GLM) to estimate the magnitude of the BOLD response at each voxel. An additional set of GLMs was computed that did not assume a shape for the HRF. This GLM was used to extract timecourses of the BOLD signal.

### 2.6 Statistical analyses

All statistical analysis, except for voxel-wise and regional ANOVAs and t-tests of fMRI data, was performed using IBM SPSS Statistics, v.20 (IBM Corporation, NY, USA). We used in-house software (FIDL) to analyze the fMRI data and results of voxel-wise statistical tests were corrected for multiple comparisons using a z-score/cluster size threshold determined from Monte Carlo simulations. Comparisons of eye data parameters and fMRI data were conducted with repeated-measures mixed model ANOVA. A sphericity correction was applied if necessary. The Independent Samples Mann–Whitney U test was used to compare means for behavioral data and the Independent Samples t-test was used to compare voxel-wise data BOLD and DTI data. Tests of normality were performed using the Shapiro–Wilk test. Statistical significance was preset at p < 0.05.

## 3 Results

### 3.1 Analysis of mTBI symptoms

**Fig. 1:**
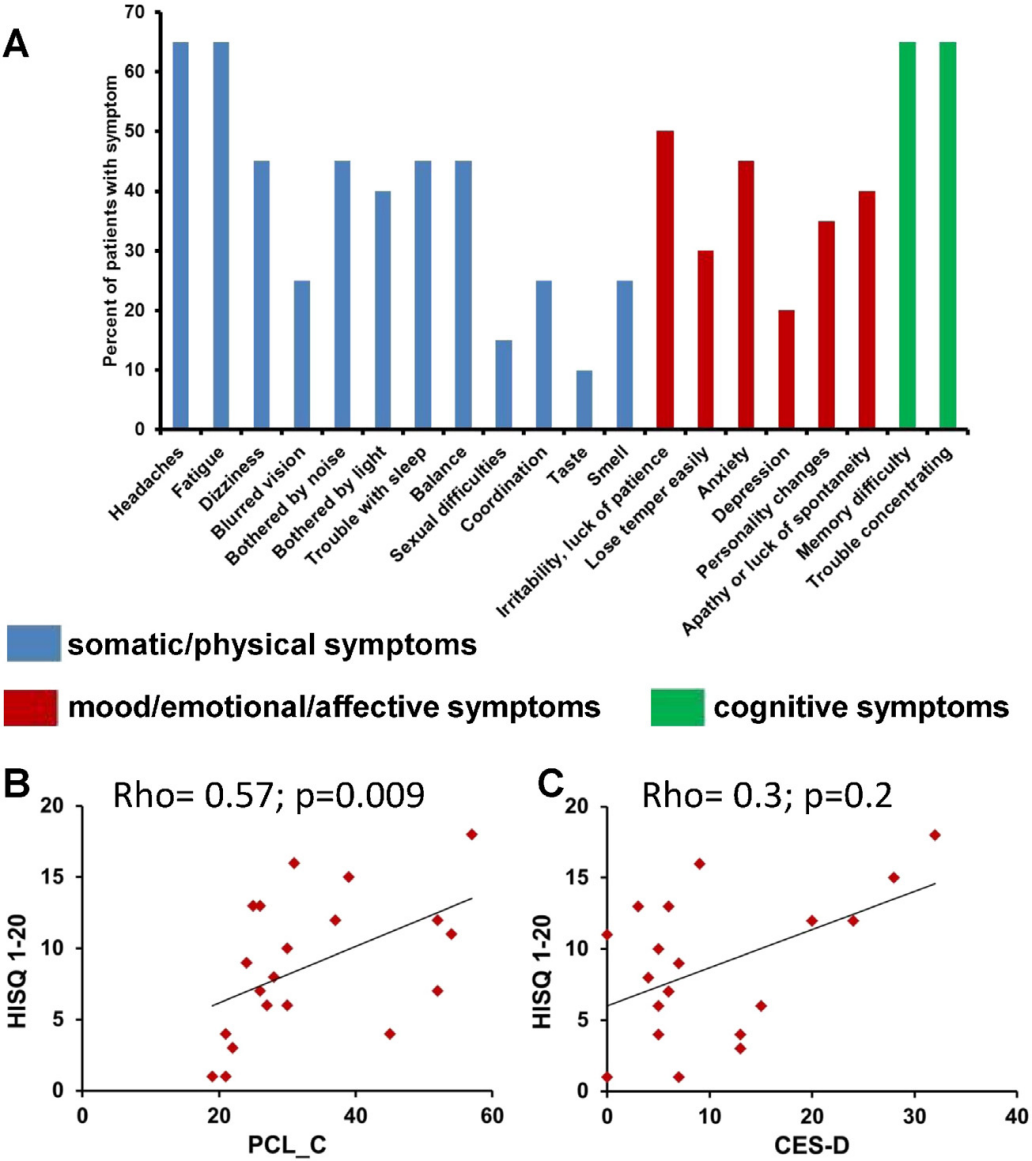
(A): Bar graph representing the percentage of mTBI patients having specific symptoms from the HISQ 1-20 questionnaire. Blue bars represent somatic/physical symptoms, red bars represent mood/emotional/affective symptoms and green bars represent cognitive symptoms. (B): PCL_C scores in mTBI patients (X axis) vs. number of reported mTBI symptoms (measured by HISQ 1-20 questionnaire) in mTBI patients (Y axis). (C): CES-D scores in mTBI patients (X axis) vs. number of reported mTBI symptoms in mTBI patients (Y axis). (For interpretation of the references to color in this figure legend, the reader is referred to the web version of this article.)

Mild TBI symptoms were classified based on the HISQ 1–20 scale. Headache is a frequently reported symptom in mTBI patients. 65% of our mTBI patients (13 out of 20) reported headaches, 40% reported sensitivity to light, 25% reported blurred vision, and 65% reported trouble concentrating, but only 20% reported depression (Fig. 1A). As expected based on the literature, there was a strong positive correlation of the PCL_C, a PTSD scale, with head injury symptoms (HISQ 1-20 (Spearman's rho = 0.57, p = 0.009; (Fig. 1B). The CES-D, a depression scale, was not significantly correlated with either HISQ 1-20 (Fig. 1C) or PCL_C scores. There was no correlation between mTBI symptoms (HISQ 1-20, PCL_C, CES-D) and months post-injury (MPI), anterograde post-traumatic amnesia (aPTA) duration, loss of consciousness (LOC) or radiological findings.

In summary the behavioral data indicate a typical distribution of mTBI symptoms with frequent reports of headache and light/noise sensitivity. Also mTBI and PTSD were more correlated with each other than with depression symptoms.

### 3.2 Analysis of resting state functional connectivity

**Fig. 2:**
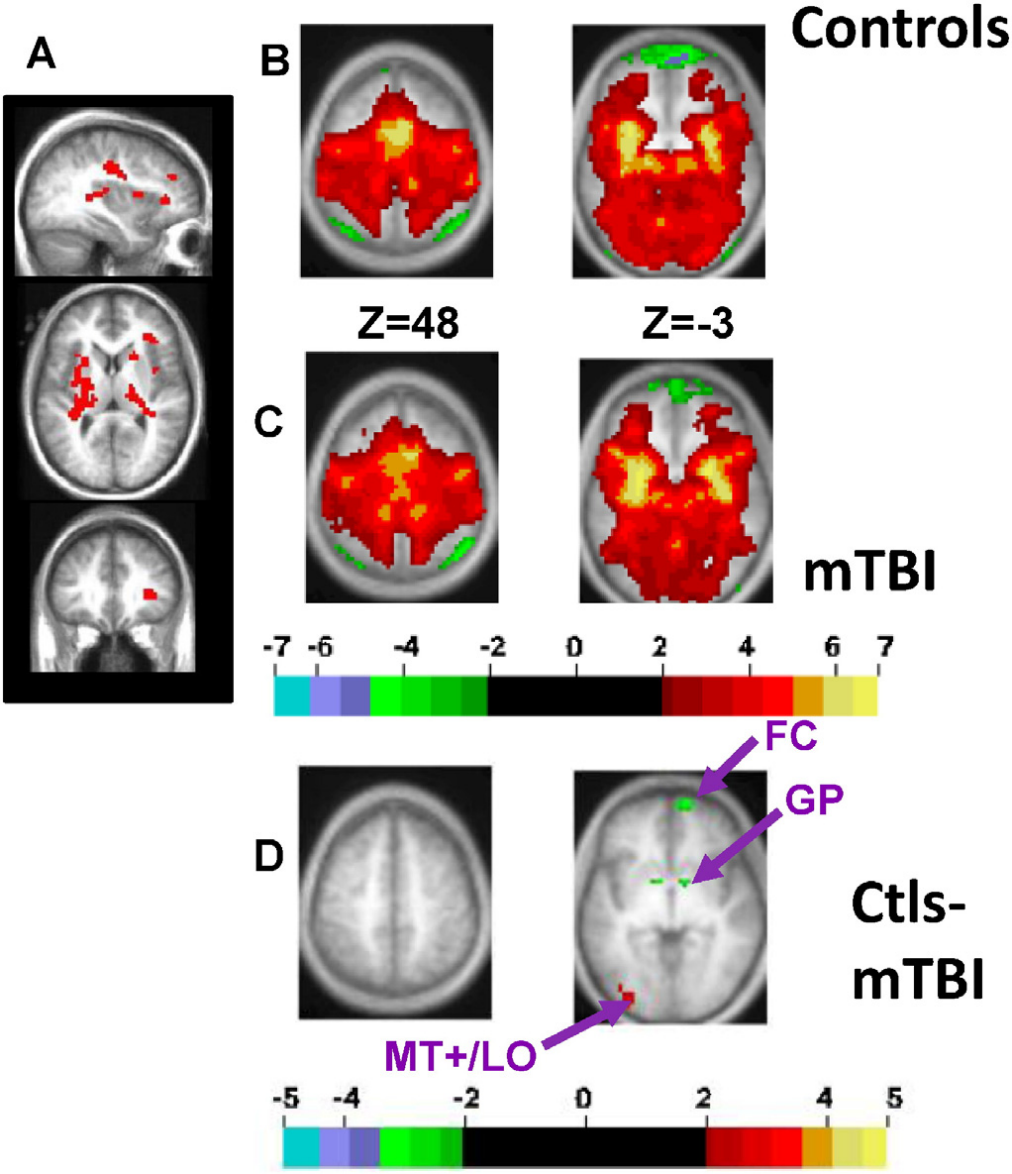
RS-fcfMRI analysis using the ‘abnormal ROI’, which showed reduced BOLD magnitudes during tracking tasks in mTBI patients relative to controls as a seed region (see text for definition). (A) The ‘abnormal ROI’. (B) Selected brain slices displaying results of a one sample t-test on rs-fcfMRI between the abnormal ROI and the rest of the brain for 21 matched control subjects, with t-values converted to z-values and corrected for multiple comparisons using a Monte Carlo correction. (C) The same analysis as (B) for 20 mTBI patients. (D) Independent unpaired t-test (with resulting z-maps corrected for multiple comparisons) between control subjects and mTBI patients. Color scale represents z-scores. (For interpretation of the references to color in this figure legend, the reader is referred to the web version of this article.)

Fig. 2A displays the ‘abnormal’ ROI from our previous study. This region includes several common (across subjects) foci in the central white matter including superior longitudinal fasciculus (SLF) and internal capsule (IC), subcortical nuclei like the basal ganglia, and some inferior frontal regions. Supplementary Fig. 1 contains a brief summary of the physiological signals previously reported in that ROI during visual tracking in mTBI patients relative to controls (Astafiev et al., 2015).

The time courses of the BOLD signal from the ‘abnormal’ ROI were significantly different between mTBI patients and controls (Supplementary Fig. 1A), but there were no significant correlations between mTBI related symptoms and BOLD magnitudes (Supplementary Fig. 1B-D) (Astafiev et al., 2015). Since these signals were recorded at the chronic stage, we posited that these regions (mainly subcortical and white matter) had been remodeled after the acute injury, and that the abnormal BOLD signal physiology reflected such remodeling. Hence we used rs-fcMRI to identify other brain regions that were functionally connected with the abnormal ROI. While rs-fcMRI is typically used to identify functional connections between brain regions, recent work indicates that it can also be used to trace connections in the white matter (Ding et al., 2013).

Fig. 2B and C show FC from the abnormal ROI to the rest of the brain in healthy controls and mTBI, while Fig. 2D shows a voxelwise t-test between the 21 matched control subjects and 20 mTBI patients. This analysis is unbiased since the abnormal ROI was determined from task scans while FC was assessed in a separate set of resting state scans. FC was in general very similar in mTBI patients and controls (Figs. 2B-C). The t-test (with resulting z-maps corrected for multiple comparisons; cluster size of 53 voxels with z ≥ 2.25), however, indicated that mTBI patients showed smaller FC with left MT+/LO (Middle Temporal and Lateral Occipital visual areas) and higher FC with left and right globus pallidus (GP) and right frontal cortex (Fig. 2D). The strength of resting FC between the abnormal ROI and the ROI in MT+/LO was not related to the presence of headache or sensitivity to light in patients.

### 3.3 Control analysis on resting state functional connectivity

On average, 83.4% of the volume of the abnormal ROI was localized to the white matter. Hence the following control analyses were restricted to the white matter component. In standard FC processing streams, ‘nuisance’ signals from a standard white matter region are often regressed out, and our initial analysis (presented in paragraph 3.2) followed this procedure. However, since we decided to focus on the FC in the white matter, white matter nuisance regression was not appropriate. Also, since FC is typically not computed using a multi-focal ROI, FC from different WM foci within the abnormal ROI were separately analyzed, without using a WM nuisance regressor. We selected the top 10 ROIs from our ‘abnormal ROI’, as determined by an automatic peak-finding algorithm, and restricted those ROIs to white matter (i.e. for each patient and control subject we selected voxels that were inside the WM for all subjects). This procedure allowed us to use the same WM ROI for each patient and control subject, eliminating the possibility that the size of the ROI affected FC. Nine of the 10 top foci contained voxels that were entirely in the WM in all patients and controls. An example of one WM ROI is presented in Supplementary Fig. 2A (“ROI”).

Notably, the FC from/to this WM ROI (“FC of ROI”) revealed connectivity with bilateral central and posterior WM similar to recently published studies using similar method (Ding et al., 2013). A voxelwise comparison of the FC for this WM ROI between mTBI patients and controls revealed an MT+/LO region similar to the ROI in Fig. 2D (yellow outline). Interestingly, all 9 WM foci taken from the ‘abnormal ROI’ showed FC with a similar MT+/LO region, as determined by the overlap of thresholded (| z | > 1.96) Z-maps (Supplementary Fig. 2B) or by the average of the same unthresholded Z-maps (not shown). Therefore, most of the major WM sub-regions of the abnormal ROI showed FC with MT+/LO.

Since regressing out a WM signal during FC preprocessing is a standard part of fcMRI processing streams, we retained the use of a WM nuisance regressor in the analyses described below. Moreover, since single WM foci inside the ‘abnormal ROI’ produced FC similar to the FC of the multifocal ROI, we present the FC analyses using the entire multifocal ‘abnormal ROI’. Use of the entire abnormal ROI may identify structures most affected by diffuse and multifocal white matter damage.

### 3.4 Analysis of task-evoked BOLD activity in visual cortex

Next, we compared task-evoked activity for patients and controls in the three regions that showed larger FC with the abnormal ROI (L and R GP, and right frontal cortex, Fig. 2D), and the one region that showed smaller FC (MT+/LO, Fig. 2D). Regional ANOVAs with the factors Task (TA, TD, GAP), Group (mTBI patients, Controls) and Time (16 MR frames) indicated that only the MT+/LO ROI (atlas coordinates x,y,z = − 35, − 84, 0) demonstrated group differences in the task-evoked BOLD response. This group difference was task specific (Group × Task × Time; F(30,1050) = 1.9, p = 0.008 after sphericity correction). MT+/LO also showed a Task × Time interaction (F(30,1050) = 3.4, p < 0.0001 corrected), indicating that activity in the region differed across tasks, but no Group × Time interaction, indicating that the overall task-evoked BOLD activity in this region was normal.

**Fig. 3:**
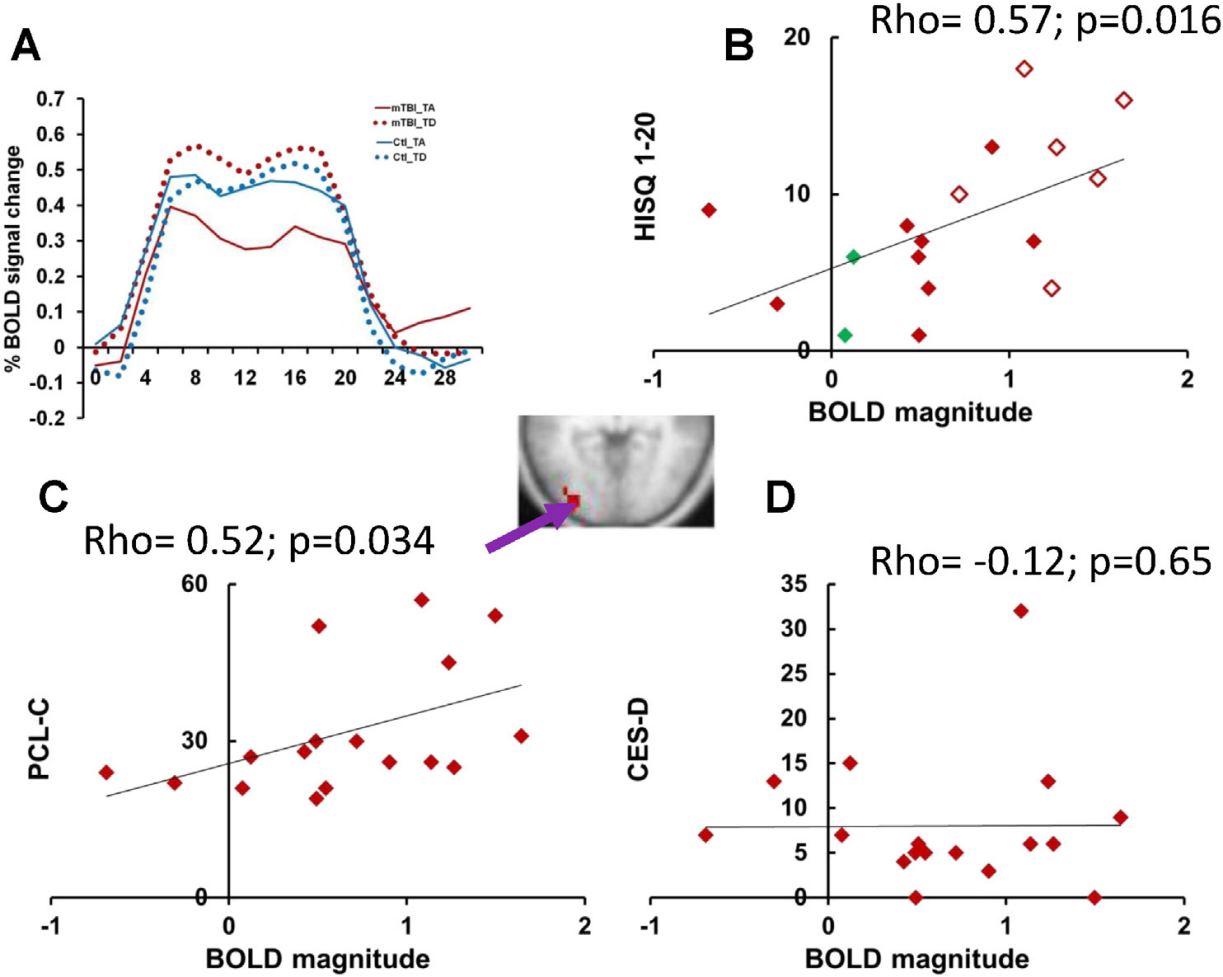
Analysis of evoked BOLD responses in visual cortex from MT+/LO ROI. (A) The time courses of the BOLD signal from the MT+/LO region (see Fig. 1D). Red lines indicate the BOLD signal in mTBI patients, blue lines in the matched control subjects. Solid lines indicate the BOLD signal during the TA task, dotted lines during the TD task. (B): BOLD magnitudes averaged across all 3 tasks from the same MT+/LO ROI (X axis) vs. the number of reported mTBI symptoms (measured by HISQ 1-20 questionnaire) in mTBI patients. mTBI patients with reported light sensitivity in HISQ 1-20 questionnaire are marked by open symbols. “Complex” mTBI patients are marked by green diamonds. (C) BOLD magnitudes averaged across all 3 tasks from the same MT+/LO ROI (X axis) vs. PCL_C scores (Y axis) in mTBI patients. (D) BOLD magnitudes averaged across all 3 tasks from the same MT+/LO ROI (X axis) vs. CES-D scores in mTBI patients. (For interpretation of the references to color in this figure legend, the reader is referred to the web version of this article.)

The time courses of the BOLD signal from the MT+/LO region (Fig. 2D and insert in Fig. 3) indicated a normally shaped BOLD response during all tracking tasks in the mTBI patients, similar to other cortical regions within the DAN and visual system (Astafiev et al., 2015). However, this region showed a larger BOLD response in patients than controls during the TD task, in which distracters were presented as well as the target, relative to the TA Task, in which only the target was presented. (i.e. in Fig. 3A, the difference between the solid (TA) and dotted (TD) lines is larger in mTBI patients (red lines) than controls subjects (blue)). This difference appeared to reflect the fact that mTBI patients showed both a smaller BOLD response in the TA task and a higher BOLD response in the TD task, relative to controls. A sub-ANOVA that compared the tracking alone and tracking with distracters tasks (Task (TA/TD), Group (mTBI patients/Controls) and Time (16 MR frames)) indicated a significant Group × Task × Time interaction (F(15,525) = 3.6, p = 0.0001 after sphericity correction) and Task × Time interaction (F(15,525) = 5.4, p < 0.0001 sphericity corrected), confirming the larger response in mTBI patients during the TD task. The larger response was not accompanied by higher distractibility, based on eye movement recordings (Astafiev et al., 2015). In particular, we did not observe significant differences in tracking accuracy after distracter presentation between mTBI patients and control subjects. Normal performance on the oculomotor task suggests that chronic mTBI patients used compensatory mechanisms and/or showed plasticity in response to injury in order to maintain performance in demanding tasks.

It is important to note that the present findings relating BOLD response magnitudes in MT+/LO to mTBI status are not inconsistent with our recently published report (Astafiev et al., 2015). In the first study the analysis was voxel-wise, not regional, and therefore much more conservative due to the need for a multiple-comparison correction. Accordingly, the MT+/LO response did not show a significant different between mTBI and healthy controls. In the current paper, the MT+/LO ROI was defined from an independent set of resting-state scans allowing a more sensitive regional analysis.

We then determined whether the task-evoked BOLD activity in the MT+/LO region was correlated with mTBI symptoms. Analysis of BOLD magnitudes in mTBI patients across all 3 tasks from the MT+/LO region revealed a significant positive correlation with mTBI symptoms (HISC 1-20; Spearman's rho = 0.57; p = 0.016; Fig. 3B), and with PTSD symptoms (PCL_C total; Spearman's rho = 0.52; p = 0.034; Fig. 3C). There was no correlation of BOLD magnitudes in mTBI patients with depression scores (CES-D; Spearman's rho = − 0.12; p = 0.65; Fig. 3D). The correlation of BOLD magnitudes from the MT+/LO region with mTBI symptoms remained significant (Spearman's rho = 0.52; p = 0.033) after regressing out the PCL_C total and CES-D scores, supporting the specificity of the association with mTBI symptoms. However, the correlation of PTSD symptoms with BOLD magnitudes in MT+/LO was not significant after regressing out HISQ and CES-D scores (Spearman's rho = .279, ns). Patients with ‘Complex mTBI’, who have positive radiological findings and/or aPTA longer than 24 h, are indicated in Fig. 3B by green diamonds. Complex mTBI patients did not have higher HISQ 1-20 scores or higher BOLD magnitudes in MT+/LO than mTBI patients.

**Fig. 4:**
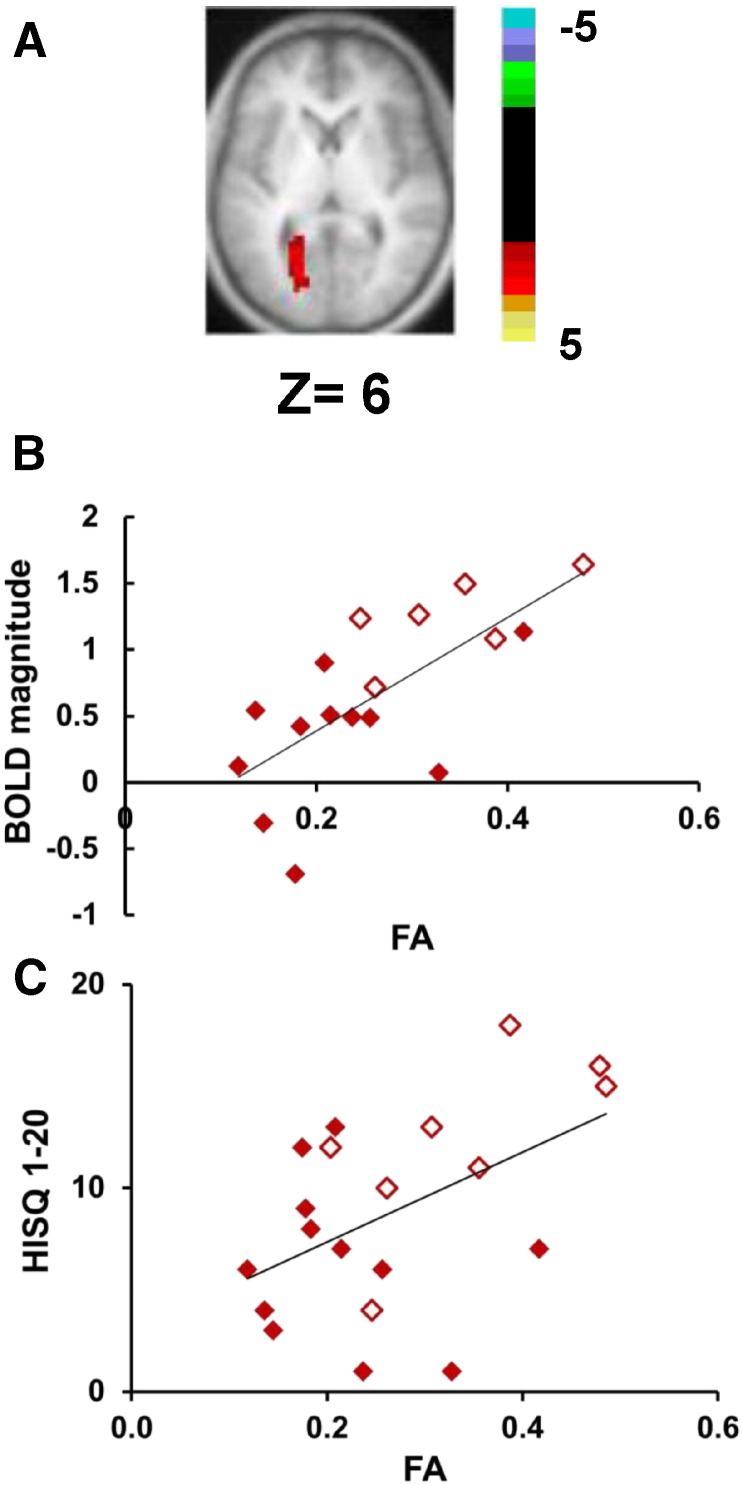
(A) Selected brain slice displaying the results of a voxel-wise correlation (corrected for multiple comparisons) of BOLD magnitudes averaged across all 3 tasks from the MT+/LO ROI (see Fig. 1D) vs. FA values. Color scale represents z-scores. (B) FA values inside the ROI in Fig. 3A (x-axis) vs. BOLD magnitudes averaged across all 3 tasks (y-axis) from the MT+/LO ROI. mTBI patients with reported light sensitivity in the HISQ 1-20 questionnaire are marked by open symbols. (C) FA values inside the ROI in Fig. 3A (x-axis) vs. the number of reported mTBI symptoms as measured by the HISQ 1-20 questionnaire in mTBI patients. mTBI patients with reported light sensitivity in the HISQ 1-20 questionnaire are marked by open symbols. (For interpretation of the references to color in this figure legend, the reader is referred to the web version of this article.)

Interestingly, mTBI patients with reported light sensitivity on the HISQ 1-20 questionnaire (Figs. 3B and 4B-C, open diamonds) had higher scores on the HISQ 1-20 questionnaire (Independent Samples Mann–Whitney U test (U test) p = 0.007), higher BOLD magnitudes in the MT+/LO region (U test p = 0.001), and higher PTSD scores (U test p = 0.007) than mTBI patients without reported light sensitivity (Fig. 3B and 4B,C, filled diamonds). These results suggest that hyper-activation of the MT+/LO region may underlie some of the visual-related symptoms of mTBI patients. Values from the CES-D scale were also higher in mTBI patients with light sensitivity, but did not reach significance (U test, p = 0.21). A comparison of patients with headache and without headache did not indicate significant differences in BOLD magnitudes from MT+/LO ROI. In summary, BOLD signals in MT+/LO showed significant differences between mTBI patients and controls, and were positively correlated with mTBI and PTSD symptoms, particularly light sensitivity.

### 3.5 Analysis of correlation of BOLD magnitudes in MT+/LO with FA

The large overlap of the abnormal ROI with white matter (Astafiev et al., 2015) and the smaller FC in mTBI patients between anterior and posterior parts of the brain, suggest that white matter was damaged in our mTBI patients, consistent with earlier DTI studies. However, we previously reported that a TBSS analysis of axial diffusivity (AD), radial diffusivity (RD) and mean diffusivity (MD) did not reveal any significant differences between mTBI patients and controls (Astafiev et al., 2015).

In order to investigate whether white matter abnormalities were related to the abnormalities of the task-evoked BOLD signal in the MT+/LO region, we conducted a voxel-wise analysis in the mTBI patients to correlate BOLD magnitudes with FA values. Because recent studies (Bouix, S., et al., 2013, Ling, J.M., et al., 2012, Lipton, M.L., et al., 2012, Mac Donald, C.L., et al., 2011, Shenton, M.E., et al., 2012, Spitz, G., et al., 2013, Strangman, G.E., et al., 2012) have reported abnormal (both increases and decreases) FA values in both gray and white matter, the analysis was not restricted to white matter voxels. Again importantly, we are not looking for mean differences in FA between patients and controls, but for regions in which the FA values correlate with the magnitude of the BOLD response in MT+/LO during visual tracking.

A significant correlation was found in an area near the left optic radiation/radiation of corpus callosum/forceps major (we will abbreviate this region as the left optic radiation (OR), Fig. 4A). A scatterplot is shown in Fig. 4B. The correlation was positive, indicating that higher FA values near the left OR were associated with higher BOLD magnitudes in left MT+/LO ROI (Spearman's rho = 0.67, p = 0.003; Fig. 4B). One interpretation of this result is that higher FA values near the left optic radiation resulted in hyperactivity of MT+/LO and higher sensitivity to light. Correspondingly, mTBI patients with light sensitivity demonstrated higher FA values in the ROI near the left OR (U test p = 0.01; Fig. 4C). However, correlations of FA values in the ROI near the left OR with the HISQ 1-20, PCL_C, and CES-D were not significant, although there was a trend for a positive correlation of FA values and HISQ 1-20 scores (Fig. 4C; Spearman's rho = .42, p = 0.068).

The conjunction of high FA values near the left optic radiation, abnormal BOLD magnitudes in MT+/LO, and light sensitivity indicate that some mTBI symptoms may be related to abnormalities in visual cortex.

## 4 Discussion

In this paper we report the results of a multimodal imaging study involving behavioral assessment, evoked and resting-state BOLD, and DTI in chronic mTBI subjects. We found that larger task-evoked BOLD activity in the MT+/LO region correlated with mTBI and PTSD symptoms, especially light sensitivity. Moreover, higher FA values near the left optic radiation (OR) were associated with both light sensitivity and higher BOLD activity in the MT+/LO region, the same region whose activity was associated with mTBI symptoms. These converging results may identify structural and physiological correlates of important symptoms following mTBI. We suggest that some PTSD and mTBI symptoms are the result of plasticity following damage to central white matter and reduced top-down control, and/or vulnerability factors that were present before the mTBI trauma, as suggested by recent studies (Davenport et al., 2014).

### 4.1 Abnormal visual cortex activity and connectivity in mTBI patients

Unfortunately, there is no accepted theory on the origin of symptoms after mTBI. The most widely accepted theory is that diffuse axonal injury causes white matter damage and disconnection of different brain regions (Hulkower, M.B., et al., 2013, Mac Donald, C.L., et al., 2011, Shenton, M.E., et al., 2012). Some studies emphasize a deficit of top-down visual attention (Corbetta and Shulman, 2002) in mTBI patients (Halterman, C.I., et al., 2006, Mayer, A.R., et al., 2012).

The most important finding of this study was the correlation between task-evoked BOLD signals during visual eye tracking in MT+/LO, which are extra-striate visual regions involved in motion and object processing, and mTBI/PTSD symptoms. The higher activity in MT+/LO also correlated with higher FA near the left OR. Notably, subjects with light sensitivity (photophobia) and accompanying headache also showed stronger BOLD responses in MT+/LO and higher FA values in the underlying white matter. Finally, mTBI patients relative to controls showed more activity in MT+/LO during the tracking task in which unexpected distracter stimuli were presented on the screen, and less activity in the tracking alone task. Although purely speculative, the difference in the sign of the BOLD changes for Tracking Along and Tracking with Distracters could reflect a loss of top-down input to MT+/LO. Lack of top-down signals might reduce the overall activity of MT+/LO (Corbetta, M. and Shulman, G.L., 2002, Serences, J.T. and Boynton, G.M., 2007), leading to a lower BOLD magnitude in the Tracking Alone Task, while at the same time increasing the sensory effects of distracters on MT+/LO activity, raising the BOLD magnitude in the Tracking with Distracters Task. Although the larger BOLD activity in the distracter task could potentially relate to the distractibility of mTBI patients, we did not find a correlation with the ‘distractibility’ item of the HISQ.

This specific set of physiological and anatomical observations relate to the frequent clinical observation that headache is a most common physical symptom after TBI, that migraine and probable migraine usually describes the majority of headaches after TBI at one year post-injury (Lucas, S., et al., 2014, Lucas, S., et al., 2012), and that headaches and light sensitivity are strongly correlated. Some researchers have proposed that cortical spreading depression (CSD) and depolarization waves, starting from visual cortex in the case of migraine, may represent a common mechanism in other brain disorders (stroke, subarachnoid hemorrhage, traumatic brain injury (Lauritzen et al., 2011)). Hence spreading depression may represent a common pathogenetic link between migraine and TBI. It is important to note that some types of headache (i.e. a tension-type headache) do not involve sensitivity to light and noise and may be linked to muscle tension and altered pain sensitivity, therefore being unrelated to visual cortex activity and CSD (Ashina et al., 2005).

However, many headaches in mTBI and migraine are triggered by sensory stimuli. Interestingly, the BOLD response to visual stimulation in primary visual cortex (V1) is greater in migraine with aura as compare to controls (Datta et al., 2013). Extrastriate visual cortex (MT+, V3A) also demonstrates higher BOLD response in migraine (Martin et al., 2011). Moreover, cortical thickness is larger bilaterally in areas involved in motion processing (V3A and MT+) in migraineurs compared with controls, in parallel with abnormal BOLD signal increases in these areas (Granziera et al., 2006). Finally, TMS studies have shown that migraine patients have a lower phosphene threshold than controls when TMS is delivered over V1 and MT+ (Aurora et al., 2003).

Based on these relationships and our results, a plausible hypothesis is that light sensitivity with accompanying headache (probably migraine) in mTBI is due to an abnormal sensitivity of motion/onset sensitive neurons in motion processing areas, which leads to an abnormally high response in MT+/LO neurons, especially to transient stimuli. In chronic patients, this sensitivity is consolidated by structural changes involving the underlying white matter. The abnormal sensitivity in motion processing regions like MT+ may be due to white matter damage in mTBI that disconnects these sensory regions from their normal top-down modulation (Corbetta, M. and Shulman, G.L., 2002, Serences, J.T. and Boynton, G.M., 2007). In fact, extra-striate visual regions are normally regulated by feedback signals that come from prefrontal and posterior parietal regions (the so called Dorsal Attention Network, DAN (Bressler, S.L., et al., 2008, Corbetta, M. and Shulman, G.L., 2002, Serences, J.T. and Boynton, G.M., 2007)), either directly or through the pulvinar. Loss of normal feedback from prefrontal and posterior parietal regions is supported in our data by the location of BOLD signal abnormalities in the dorsal white matter. These abnormalities occurred in regions corresponding to the superior longitudinal fasciculus (SLF), which connects dorsal occipital and posterior parietal to prefrontal regions, as recently reported (Astafiev et al., 2015). Our hypothesis is consistent with prior work showing that top-down attention deficits, as measured with the executive component of the Attention Network Test (ANT), persist throughout the first month post-injury (Halterman et al., 2006). Deficits of top-down visual attention have been reported to accompany abnormal activation of left visual cortex near optic radiation (Mayer et al., 2012). Alternatively, as suggested by a reviewer, the MT+/LO region might be sensitive to mTBI damage due to its heavy myelination, reflecting the importance of white matter abnormalities in mTBI (Armstrong et al., Epub ahead of print).

Our control analysis revealed that different parts of the WM have reduced FC with the MT+/LO ROI in mTBI patients. This fact is surprising, but is partially supported by a previous study (Boes et al., 2015), which demonstrated that distributed lesions in subcortical structures and white matter may involve abnormal FC in regions close to the MT+/LO ROI. It is widely accepted that signal changes in WM and CSF represent primarily non-neuronal fluctuations (i.e. scanner instabilities, subject motion and physiological artifacts including respiration and cardiac effects (Dagli, M.S., et al., 1999, Windischberger, C., et al., 2002)). On the other hand, some researchers report that the power of temporal variations of low frequency BOLD oscillations in WM is about 80% of BOLD oscillations in gray matter (Ding et al., 2013). Also, despite the fact that blood flow in WM is about 25% of that in gray matter, the oxygen extraction rates are similar (Raichle et al., 2001). Moreover, as we demonstrated before (Astafiev et al., 2015), the shape of the evoked BOLD response is similar in white and gray matter for healthy control subjects (see also Supplementary Figs. 1A and 3A).

Our control analysis suggests that FC based on BOLD oscillations in WM can be a useful tool for studying mTBI related abnormalities. But our results should be validated in an independent study of FC in white matter (Ding et al., 2013).

### 4.2 Brain correlates of mTBI symptoms

Both PTSD and mTBI symptoms were associated with higher BOLD magnitudes in the MT+/LO region, but little relationship was seen with depression symptoms. Some studies have reported larger BOLD signals in visual cortex, including an area near LO, in veterans with PTSD (Hendler et al., 2003) and/or mTBI (Scheibel et al., 2012), in line with our findings. PTSD and mTBI are widely thought to be closely linked. mTBI is more frequently associated with PTSD than any other type of injury (Hoge et al., 2008), and PTSD is the strongest predictor of post-concussion syndrome after mTBI (Schneiderman et al., 2008).

Future longitudinal multimodal imaging studies will be necessary to fully understand the link between BOLD activity, DTI parameters and mTBI/PTSD symptoms in mTBI patients.

## 5 Study limitations

The main limitation of this study is the relatively small sample size. Our sample was comparable to that of many studies in the literature. Also, the level of significance in the voxel-wise statistical maps was corrected for multiple comparisons at the whole brain level following a random effect statistical model that allowed for generalization at the population level. Nevertheless, this study will require replication and validation in a separate and larger independent sample. Because the MT+/LO region was originally identified from a functional connectivity analysis, the subsequent analysis of evoked BOLD differences between mTBI patients and controls was conducted regionally. However, we acknowledge that these group differences in evoked BOLD activity did not survive a whole-brain correction for multiple comparisons (Astafiev et al., 2015), highlighting the importance of replicating this result in an independent and larger sample.

The sample contained three patients with PTA longer than 24 h, and three patients with radiological abnormalities, of whom 2 possessed both characteristics, resulting in a total of four ‘more severe’ patients. These patients are defined today as ‘complex mTBI’ (Dempsey, K.E., et al., 2009, Lewine, J.D., et al., 2007). Their enrollment was due to the use of inclusion criteria that were more liberal than current standards when the Brain Trauma Foundation, UCSD, and Washington University joined in 2009 in a consortium under whose auspices this research was performed. However, even though all 4 complex mTBI subjects participated in psychometric/visual tracking testing and FC/DTI session, only two of these patients (indicated by green diamonds in Fig. 3B) underwent task fMRI. Therefore it is unlikely that the correlation of BOLD magnitudes with symptoms was related to this factor.

However, to further address this issue, we recomputed all of the regional analyses after eliminating the 4 complex mTBI subjects. All results remained significant, except for the correlation of BOLD activity in the MT+/LO region with PCL_C total (the correlation was marginal, Spearman's rho = 0.48; p = 0.068).

Another limitation is the use of a multifocal ROI as a seed region in the functional connectivity analyses, since the resulting connectivity values were not localized to pairs of regions. However, our control analysis demonstrated that the major WM foci within this ROI in fact showed functional connectivity with the MT+/LO region that was the focus of the paper.

The following are the supplementary data related to this article.

Supplementary Fig. 1 Analysis of evoked BOLD responses from the ‘abnormal’ ROI. (A): The time course of the BOLD signal in the ‘abnormal’ ROI. The canonical hemodynamic response function (HRF) used in the analysis to compute the BOLD magnitudes is also shown (labeled “canonical response”). (B): BOLD magnitudes averaged across all 3 tasks from the same ‘abnormal’ ROI (X axis) vs. number of reported mTBI symptoms (measured by HISQ 1-20 questionnaire) in mTBI patients. (C): BOLD magnitudes averaged across all 3 tasks from the same ‘abnormal’ ROI (X axis) vs. PCL_C scores (Y axis) in mTBI patients (red diamonds) and matched control subjects (blue circles). (D): BOLD magnitudes averaged across all 3 tasks from the same ‘abnormal’ ROI (X axis) vs. CES-D scores in mTBI patients (red diamonds) and matched control subjects (blue circles).

Supplementary Fig. 2 MT+/LO voxels are consistently found in FC maps from single white matter foci within the ‘abnormal ROI’. (A): A transverse slice showing one of the white matter foci (‘ROI’) that was inside the multifocal ‘abnormal ROI’. This focus was used as a seed in an FC analysis. FC maps were generated for each subject and a one-sample voxelwise t-test was conducted on the maps, followed by a correction for multiple-comparisons. A slice from the multiple-comparison corrected map is shown (“FC of ROI”). On the right are three transverse slices displaying an uncorrected z-map (thresholded at | z |>1.96) based on an unpaired t-test contrasting the FC maps from control subjects and mTBI patients using the “ROI” as a seed region. The yellow line outlines the MT+/LO region. The white matter signal was not regressed during pre-processing. (B): Three transverse slices displaying the overlap of nine FC difference maps (mTBI vs controls) generated using the top 9 white matter foci of the ‘abnormal ROI’ as seeds. Each difference map was thresholded at | z |>1.96 and converted into binary mask. The yellow line outlines the MT+/LO region. Voxels with 100% overlap are in red. One or more voxels from the MT+/LO ROI were present in all of the maps.

## Funding

This work was supported by a James S. McDonnell Foundation grant to the Attention Dynamics Consortium in Traumatic Brain Injury (ADC-TBI) and by the National Institute of Neurological Disorders (NINDS) grant R01 NS095741. Serguei V. Astafiev, PhD was partially supported by funds from the Department of Neurology, Washington University in St. Louis.

## Author disclosure statement

No competing financial interests exist.
