# Supplementary figures and images for: Exploring the physiological correlates of chronic mild traumatic brain injury symptoms
Source: Neuroimage Clin. 2016 Jan 6;11:10–9. doi: 10.1016/j.nicl.2016.01.004 (PMC4732189; doi:10.1016/j.nicl.2016.01.004)

**A**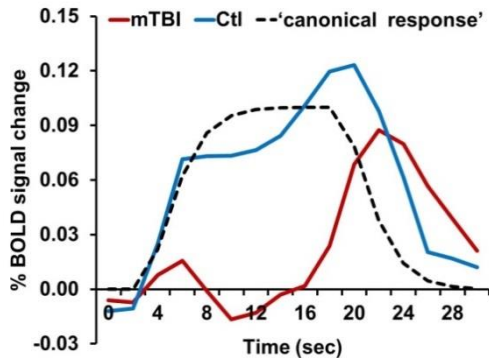**B**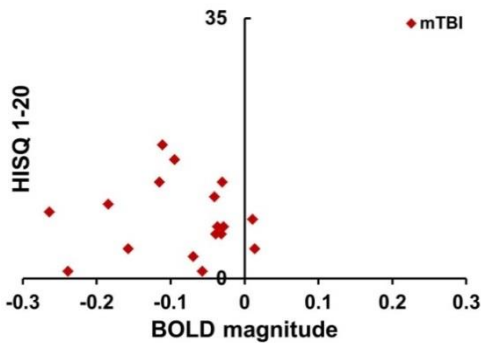**C**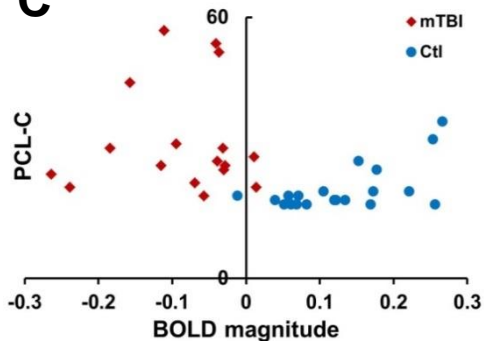**D**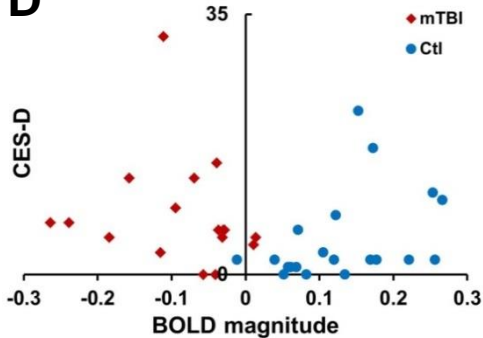

Supplement: Supplementary Fig. 1 — Analysis of evoked BOLD responses from the ‘abnormal’ ROI. (A): The time course of the BOLD signal in the ‘abnormal’ ROI. The canonical hemodynamic response function (HRF) used in the analysis to compute the BOLD magnitudes is also shown (labeled “canonical response”). (B): BOLD magnitudes averaged across all 3 tasks from the same ‘abnormal’ ROI (X axis) vs. number of reported mTBI symptoms (measured by HISQ 1-20 questionnaire) in mTBI patients. (C): BOLD magnitudes averaged across all 3 tasks from the same ‘abnormal’ ROI (X axis) vs. PCL_C scores (Y axis) in mTBI patients (red diamonds) and matched control subjects (blue circles). (D): BOLD magnitudes averaged across all 3 tasks from the same ‘abnormal’ ROI (X axis) vs. CES-D scores in mTBI patients (red diamonds) and matched control subjects (blue circles). [file mmc1.pdf]

**A)** FC of single WM foci inside abnormal ROI

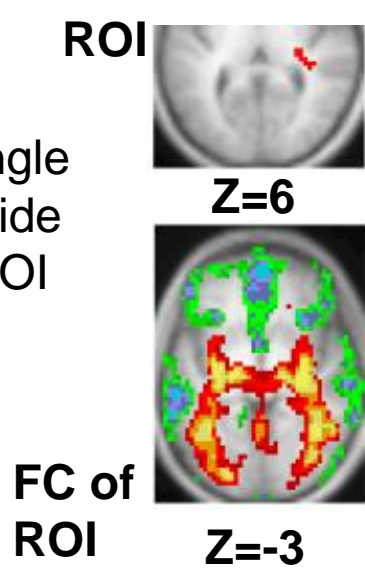

**Ctls-mTBI**

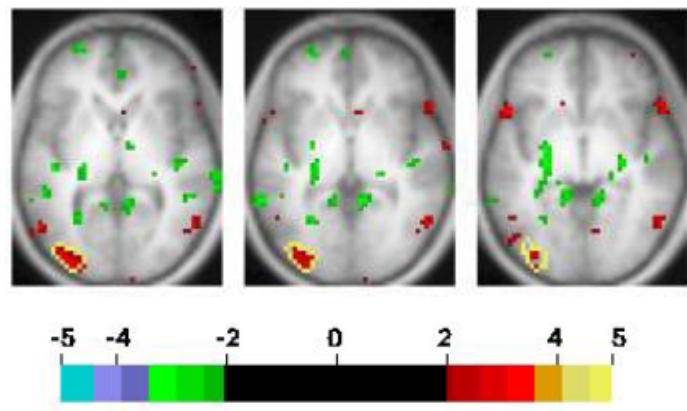

**B)** Overlap of nine FC difference maps

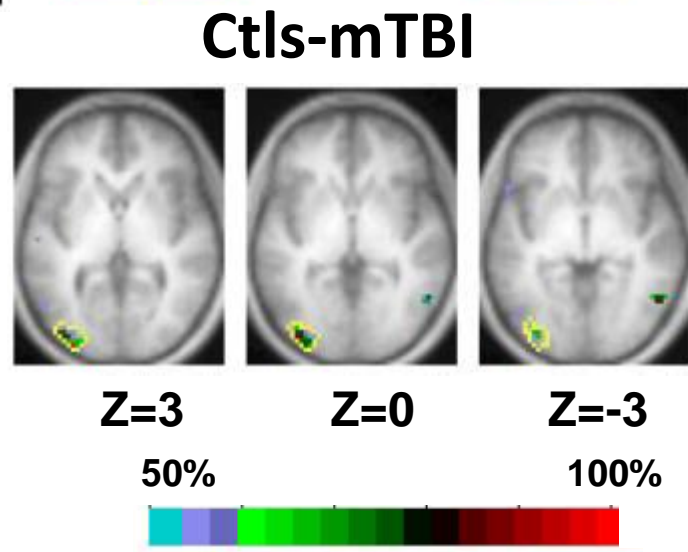

Supplement: Supplementary Fig. 2 — MT+/LO voxels are consistently found in FC maps from single white matter foci within the ‘abnormal ROI’. (A): A transverse slice showing one of the white matter foci (‘ROI’) that was inside the multifocal ‘abnormal ROI’. This focus was used as a seed in an FC analysis. FC maps were generated for each subject and a one-sample voxelwise t-test was conducted on the maps, followed by a correction for multiple-comparisons. A slice from the multiple-comparison corrected map is shown (“FC of ROI”). On the right are three transverse slices displaying an uncorrected z-map (thresholded at | z |>1.96) based on an unpaired t-test contrasting the FC maps from control subjects and mTBI patients using the “ROI” as a seed region. The yellow line outlines the MT+/LO region. The white matter signal was not regressed during pre-processing. (B): Three transverse slices displaying the overlap of nine FC difference maps (mTBI vs controls) generated using the top 9 white matter foci of the ‘abnormal ROI’ as seeds. Each difference map was thresholded at | z |>1.96 and converted into binary mask. The yellow line outlines the MT+/LO region. Voxels with 100% overlap are in red. One or more voxels from the MT+/LO ROI were present in all of the maps. [file mmc2.pdf]
